# *Gllac7* Is Induced by Agricultural and Forestry Residues and Exhibits Allelic Expression Bias in *Ganoderma lucidum*

**DOI:** 10.3389/fmicb.2022.890686

**Published:** 2022-06-30

**Authors:** Lining Wang, Xiaoxia Ding, Qinghua Huang, Biao Hu, Lei Liang, Qingfu Wang

**Affiliations:** ^1^Guangdong Engineering Laboratory of Biomass High-Value Utilization, Guangdong Plant Fiber Comprehensive Utilization Engineering Technology Research and Development Center, Guangzhou Key Laboratory of Biomass Comprehensive Utilization, Institute of Biological and Medical Engineering, Guangdong Academy of Sciences, Guangzhou, China; ^2^Key Laboratory of Quality Evaluation of Chinese Medicine of the Guangdong Provincial Medical Products Administration, the Second Clinical College, Guangzhou University of Chinese Medicine, Guangzhou, China

**Keywords:** *G. lucidum*, laccase, agricultural and forestry residues, transcriptome profile, allelic expression bias, zinc finger

## Abstract

*Ganoderma lucidum* has a wide carbon spectrum, while the expression profile of key genes relevant to carbon metabolism on different carbon sources has been seldom studied. Here, the transcriptomes of *G. lucidum* mycelia cultured on each of 19 carbon sources were conducted. In comparison with glucose, 16 to 1,006 genes were upregulated and 7 to 1,865 genes were downregulated. Significant gene expression dynamics and induced activity were observed in laccase genes when using agricultural and forestry residues (AFRs) as solo carbon sources. Furthermore, study of laccase gene family in two haploids of *G. lucidum* GL0102 was conducted. Totally, 15 and 16 laccase genes were identified in GL0102_53 and GL0102_8, respectively, among which 15 pairs were allelic genes. Gene structures were conserved between allelic laccase genes, while sequence variations (most were SNPs) existed. Nine laccase genes rarely expressed on all the tested carbon sources, while the other seven genes showed high expression level on AFRs, especially *Gllac2* and *Gllac7*, which showed 5- to 1,149-fold and 4- to 94-fold upregulation in mycelia cultured for 5 days, respectively. The expression of *H53lac7* was consistently higher than that of *H8lac7_1* on all the carbon sources except XM, exhibiting a case of allelic expression bias. A total of 47 SNPs and 3 insertions/deletions were observed between promoters of *H53lac7* and *H8lac7_1*, which lead to differences in predicted binding sites of zinc fingers. These results provide scientific data for understanding the gene expression profile and regulatory role on different carbon sources and may support further functional research of laccase.

## Introduction

*Ganoderma lucidum* is a typical white-rot fungus and has been suggested as a potential model system for medicinal mushroom study (Chen et al., 2012). *G. lucidum* has rich pharmacological ingredients, and has been used for 1000 years (Bishop et al., 2015). Traditional cultivation of *G. lucidum* on basswood, results in a large demand for forestry resources. Thus, new alternatives of biomass resources need to be discovered to improve the current situation and promote the sustainable development of *G. lucidum* industry. To date, sawdust cultivation and substituted cultivation using AFRs (lignocellulosic compounds, such as cotton seed husk, corn cobs, wheat-straw, poplar wood chip, wheat bran, and date palm leaf; Hapuarachchi et al., 2018; Kurd-Anjaraki et al., 2022) and even food or diaper waste-based cultivations have been achieved (Khoo et al., 2022).

To colony on those substrates, several secreted extracellular enzymes such as laccase, participate in degradation of lignocellulose (especially lignin) to nutrients which suitable for mycelial growth of *G. lucidum* (Zhou et al., 2018; Yuliana et al., 2020). Besides, laccases are involved in primordium formation and fruiting body development of fungi (Das et al., 1997; Lu et al., 2015; Jiao et al., 2018; Jin et al., 2018). Laccase (EC 1.10.3.2) is a group of phenol oxidases that belongs to the family of multi-copper oxidases. It usually contains three cupredoxin-like domains and is widely distributed in fungi, bacteria, and insects (Giardina et al., 2010; Hakulinen and Rouvinen, 2015). Laccases are multipurpose biocatalysts widely applied in industrial process (Virk et al., 2012; Rodríguez-Delgado et al., 2015; Teng et al., 2019; Backes et al., 2021). The majority of the characterized laccases so far are derived from white-rot fungi. Laccase derived from *G. lucidum* shows strong temperature and pH tolerance, as well as excellent performance on dye decolorization (Palazzolo et al., 2019), bioethanol production (Fang et al., 2015), lignocellulose degradation (Sitarz et al., 2013), and phenols degradation etc. (Brugnari et al., 2021). Additionally, heterologous expression of *G. lucidum* laccase in *Pichia pastoris* has been used for large-scale commercial production (You et al., 2013).

Evidences have shown that the production and activity of laccase are closely related to substrate constitutes (nutrient levels), and lignocellulosic compounds such as corncob, rice/wheat-straw, and even municipal food waste (Wang et al., 2015a; Gupta and Jana, 2019; Yuliana et al., 2020) can promote the production of *G. lucidum* laccase. Besides, the production and activity of *G. lucidum* laccase can be induced by exogenous additives (such as Cu^2+^, kraft lignin, ferulic acid, phenolic, glycerol and ethanol) and co-cultivation with other fungi (Li et al., 2011; Manavalan et al., 2013; Kuhar and Papinutti, 2014; Kuhar et al., 2015; Rodrigues et al., 2019). According to previous researches, the *G. lucidum* genome encoded 13 or 16 laccase genes in different strains (Chen et al., 2012; Liu et al., 2012), and different isozymes may have different expression patterns or differentially contribute to total laccase activity in *Ganoderma* species (Zhou et al., 2018; Qin et al., 2019; Ho et al., 2020). Studies have indicated that the expression of laccase is regulated by TFs (Polanco et al., 2006; Qi et al., 2017; Zhang et al., 2021). Moreover, allelic expression bias was reported in fungi (Ha et al., 2017; Gehrmann et al., 2018; Wang et al., 2021). To date, few studies on the regulation of laccase have been conducted and whether there is allelic expression bias in *Ganoderma* laccase has not been revealed.

AFRs, rich in lignocellulose consisting of lignin, cellulose, and hemicellulose, are misplaced resources. In this study, to investigate the utilization of *G. lucidum* mycelia of these carbon sources and to discover the key genes involved in the process, the mycelia of *G. lucidum* strain GL0102 was cultured on various carbon sources (19 kinds) and transcriptome sequencing were conducted. And the laccase gene family showing impressive performance in the transcriptome analysis was systematically studied based on two haploid genomes derived from two mating-compatible monokaryon strains. The findings here shed light on the gene expression and regulation of laccase that play important role in carbon utilization in *G. lucidum*.

## Materials and Methods

### Strains and Cultivation

The dikaryotic *G. lucidum* strain GL0102 (the same strain with “Zhi 102” of Mycological Research Center, Fujian Agriculture and Forestry University) was maintained on Difco^™^ Potato Dextrose Agar (PDA) at 4°C and stored at the Institute of Biological and Medical Engineering, Guangdong Academy of Sciences. Minimal medium plates containing glucose (20 g/l, MM), (NH4)_2_SO_4_ (1.5 g/l), K_2_HPO_4_ (1.0 g/l), MgSO_4_ (0.3 g/l), vitamin B1 (0.5 mg/l), and agar (18 g/l) were prepared. Then, the glucose was replaced with an equal amount of 17 kinds of other carbon sources (20 g/l, Table 1) to make media with different carbon sources. Specifically, the AFRs (such as rice-straw and wheat-straw) were dried and crushed using a pulverizer, and were added to 500 ml triangular flasks together with other components of the media. One rotor was put in each triangular flask before sterilization. Then the triangular flasks were stirred on a magnetic stirrer for 20 s. The media (<50°C) were poured rapidly into the plates (9 cm in diameter), and stirred for 3 s after each pouring to ensure the homogeneity of the medium. Besides, to speed up the solidification and avoid settling of insoluble components, the plate cover was open and the media was blown by sterile air. The medium preparation ensured maximal contact of the mycelia with the carbon sources. GL0102 was then inoculated into the above culture media and PDA plates and cultured at 28°C for 5 days. For media with MM, lignin (LM) and cellulose (CM), 4 mycelial blocks were inoculated in each plate, and 60 plates in total; for media with other carbon sources, one mycelial block was inoculated in each plate, and 30 plates in total. For each sample, the mycelia (approximately 10–20 plates) were quickly scraped and mixed to produce a biological replicate, which was then frozen in liquid nitrogen and stored at −80°C. Three replicates were prepared for each of the treatments. Besides, GL0102 mycelia cultured on the following six carbon sources for 3 days and 7 days were also collected in the same way: glucose, lignin, cottonseed hull, rice-straw, bagasse, and PDA. The growth rates of GL0102 on different carbon sources were determined at 28°C based on the hyphal radical length.

**Table 1:** Carbon sources used in this study.

**Carbon source**	**Abbreviation**	**Origin**	**Scientific name**	**Part**
Glucose	MM	Sigma-Aldrich, America	–	–
Lignin	LM	Sigma-Aldrich, America	–	–
Cellulose	CM	Sigma-Aldrich, America	–	–
Xylan	XM	Sigma-Aldrich, America	–	–
Corncob	COM	Dezhou, Shandong Province	*Zea mays* L.	–
Cottonseed hull	CHM	Dezhou, Shandong Province	*Gossypium hirsutum* L.	Seed coat
Wheat-straw	WSM	Dezhou, Shandong Province	*Triticum aestivum* L.	Stem
Rice-straw	RSM	Ganzhou, Jiangxi Province	*Oryza sativa* L.	Stem
Bran	BRM	Ganzhou, Jiangxi Province	*O. sativa* L.	–
Pine wood sawdust	PWM	Ganzhou, Jiangxi Province	*Pinus massoniana* Lamb.	Stem
Pine needle	PNM	Ganzhou, Jiangxi Province	*P. massoniana* Lamb.	Leaf
Spruce wood sawdust	SWM	Ganzhou, Jiangxi Province	*Cunninghamia lanceolata* (Lamb.) Hook.	Stem
Spruce needle	SNM	Ganzhou, Jiangxi Province	*C. lanceolata* (Lamb.) Hook.	Leaf
Pteridophyte	PTM	Ganzhou, Jiangxi Province	*Dicranopteris pedate* (Houttuyn) Nakaike	Stem, leaf
Chestnut shell	CSM	Heyuan, Guangdong Province	*Castanea mollissima* Blume	Pericarp
Chestnut leaf	CLM	Heyuan, Guangdong Province	*C. mollissima* Blume	Leaf
Bagasse	BM	Sanya, Hainan Province	*Saccharum officinarum* L.	Stem
Oak wood sawdust	OWM	Wuhan, Hubei Province	*Quercus variabilis* Blume	Stem
Potato dextrose agar	PDA	Difco^™^ PDA, BD, America	–	–

### RNA-Sequencing and Expression Profile Analysis

RNA-Seq was performed from mycelia cultured on the above 19 different conditions for 5 days, each in triplicate. Extraction and quality control of the total RNA from each sample were conducted using methods previously reported (Wang et al., 2018), and RNA with RNA Integrity Number ≥ 7.5 was kept (Supplementary Table 1). cDNA library construction and sequencing were carried out following protocols of MGI sequencing platform and paired-end 150-bp reads were generated. Raw read quality was assessed using FastQC^1^, and low-quality bases or reads were filtered out using Skewer (Jiang et al., 2014) with parameters: “-q 20 -Q 30 -l 50” (Supplementary Table 2). Expression levels (transcript per million, TPM) of genes in mycelia cultured on different carbon sources were calculated using HISAT2 and StringTie (Pertea et al., 2016). Two reference haploid genomes, GL0102_8 and GL0102_53 (two mating-compatible monokaryons from GL0102)^2^ were downloaded from GPGD (Liao et al., 2021). Hierarchical clustering analysis of TPM values was performed using the “pheatmap” package^3^ in R. Differentially expressed genes (DEGs) were identified using the “DESeq2” package (Wang et al., 2010), using genome of GL0102_8 as the reference. DEGs were classified as genes that had log_2_FoldChange ≥2 and value of *p* ≤1e-6.

### Gene Functional Enrichment Analysis

Gene ontology (GO) enrichment analysis was carried out using clusterProfiler (Yu et al., 2012), and enrichment results with a value of *p* <1 × 10^−3^ were retained.

### Measurement of Laccase Activity

Ground powder (0.2 g) from mycelia in the presence of liquid nitrogen was extracted using 800 μl of 0.9% normal saline at 4°C for 10 min. After centrifuging (10,000 × g, 15 min, 4°C), the supernatant was used as the crude enzyme. The enzymatic activity of laccases was assayed by the ABTS (2, 2′-azino-bis-3-ethylbenzthiazoline-6-sulphonate) method (Johannes and Majcherczyk, 2000) using the following steps: 250 μl enzyme solution was added to the mixture of 500 μl acetate buffer (pH 4.5) and 250 μl ABTS (0.5 mm), then the absorbance at 420 nm was recorded within 3 min. One unit of enzyme activity was defined as the amount of laccase that oxidized 1 μm ABTS per minute.

### Identification of Laccase Genes

Firstly, all the annotated proteins in GL0102_8 and GL0102_53 genomes were searched against the PFAM database (Pfam 32.0) using PfamScan (E value ≤1e-5).^4^ Genes with hits to PFAM ID PF00394.24, PF07731.16, and PF07732.17 were considered as the candidate laccase genes. Secondly, the genes were viewed and corrected using the Apollo browser (Dunn et al., 2019) as previously described (Wang et al., 2020). Thirdly, to rule out the false-positive results, all the laccase genes were reconfirmed using PfamScan. Finally, the whole set of laccase genes of GL0102_8 was searched against that of GL0102_53 using BlastP (Camacho et al., 2009). The allelic (one-to-one) relationship among these laccase genes was confirmed according to the amino acid (aa) similarity (identity ≥95%). For the other three individuals of *G. lucidum*, namely CGMCC5.0026 (Chen et al., 2012), P9 (single basidiospore; Liu et al., 2012), and Lingjian-2 (Tian et al., 2021), the laccase genes was identified following the same process above.

### Sequence Information Analysis and Duplication Events Identification

The number of aa, isoelectric point (pI), and molecular weight (MW) were computed using ProtParam.^5^ The presence of signal peptide cleavage sites was obtained using SignalP 5.0.^6^ The domains of laccases were identified using PfamScan.^7^ The duplication of laccase genes was identified by the two criteria: (1) the protein length of the shorter sequence covered ≥75% of the longer sequence and (2) the similarity of the two aligned sequences was ≥90%.

### Phylogenetic Analysis of Laccase Genes

A multiple sequence alignment of laccase genes in GL0102_8 and GL0102_53 was performed on full-length protein sequences using clustal (Sievers et al., 2011). A maximum likelihood (ML) phylogenetic tree was constructed using RAxML (Stamatakis, 2014) with 1,000 bootstrap replicates. In addition, a ML phylogenetic tree containing laccase protein sequences in three additional *G. lucidum* individuals (CGMCC5.0026, P9, and Lingjian-2) was built following the same process.

### Genetic Variation Detection and SNP Validation Between Paired Allelic Laccase Genes

Genetic variations were detected using MEGA7 (Kumar et al., 2016) after alignment using clustal, and the exhibition of SNPs were plotted using trackViewer (Ou and Zhu, 2019). To validate the SNP loci, the cDNA derived from mycelia cultured on RSM was used as PCR amplification template. The synthesizing of primers (Supplementary Table 3), cloning and sequencing of amplification products were conducted by Tsingke Biotechnology Co., Ltd.

### Expression Analysis by Quantitative PCR

RNA samples for sequencing were further synthesized into cDNA. Glyceraldehyde 3-phosphate dehydrogenase (*gapdh*) was used as a reference. Primers for the laccase genes (Supplementary Table 3) were designed and synthesized by Tsingke Biotechnology Co., Ltd. The qPCR reaction was performed using the Applied Biosystems ABI 7500 PCR System (ABI, United States) according to previously reported methods (Wang et al., 2017). The PCR amplification mixture contained 1 μl of cDNA, 10 μl of ChamQ Universal SYBR qPCR Master Mix (Vazyme Biotech Co., Ltd), 0.4 μl of 10 μm forward and reverse primers, and 8.2 μl ddH_2_O. The PCR reaction was performed with the initial denaturation step for 3 min at 95°C; 40 cycles of 3 s at 95°C and annealing at 60°C for 32 s, and a holding step for 30 s at 72°C. The melting curve (60–95°C) and agarose gel electrophoresis of qPCR products were used to check the specificity of each qPCR reaction. The standard curves were generated using a twofold dilution gradient of the cDNA. Amplification efficiencies (E = 10^-1/slope^–1) and correlation coefficient (R^2^) values were calculated by standard curves. The relative gene expression was calculated with the 2^–ΔΔC^_T_ method (Livak and Schmittgen, 2001). Parametric one-way analysis of variance (ANOVA) followed by Duncan’s test was used to calculate significant differences among different groups (*p* < 0.05).

### Detection and Validation of Allelic Expression Bias

The allelic expression bias was identified based on RNA-Seq read depths of SNP loci between alleles. The validation of allelic expression bias was conducted as follows: (1) primers were designed at flanking regions of SNP locus (Supplementary Table 3); (2) PCR amplification was conducted using cDNA derived from mycelia cultured on RSM; and (3) PCR products was cloned and then randomly selected and sequenced at Tsingke Biotechnology Co., Ltd.

### Co-expression Analysis of Laccase Genes

Co-expression analysis was conducted by Multiscale Embedded Gene Co-expression Network Analysis (MEGENA) pipeline (Song and Zhang, 2015) as follows: (1) pairwise Pearson correlation coefficients of all the DEGs were calculated based on expression TPM values; (2) planar filtered network was set up by taking significant correlation pairs; and (3) multiple correspondence analysis was employed to cluster genes into modules.

### Promoter Sequence Analysis and Prediction of Binding Sites

The 3 kb upstream of the start codon of laccase was extracted as the promoter region. Promoter sequences were aligned and analyzed by MEGA7. The binding sites matrix of fungi were downloaded from JASPAR^8^, and CiiiDER^9^ were used to predict the possible binding sites.

## Results

### Mycelia Cultured on Different Carbon Sources and Gene Expression Pattern

**Figure 1:**
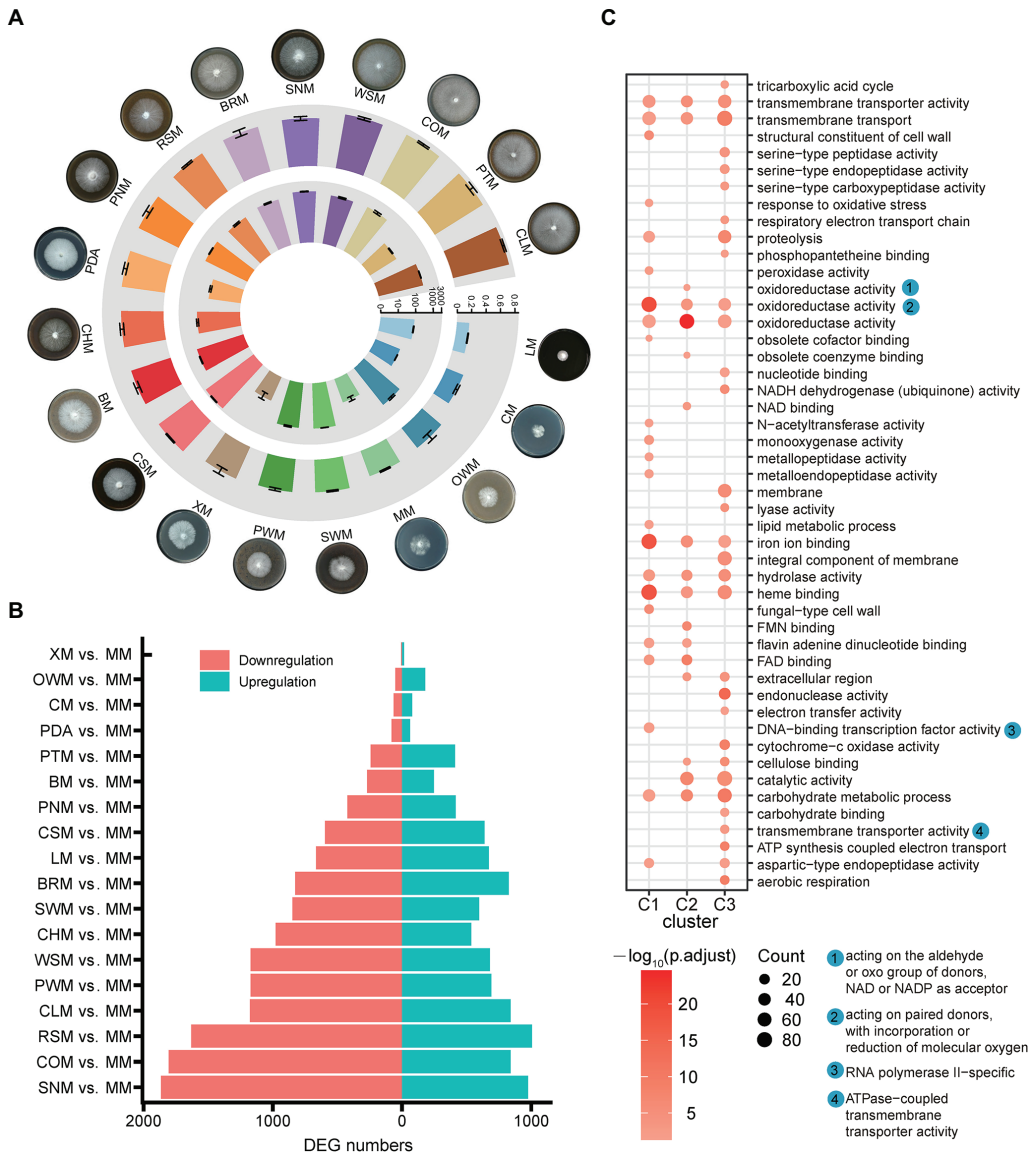
*Ganoderma lucidum* mycelia cultured on different carbon sources and differentially expressed genes. **(A)** Growth status of mycelia (outer circle), growth rate of mycelia (middle circle, cm/d), and laccase activity of mycelia (inner circle, U/mg). **(B)** DEGs in the comparison of MM with all the other carbon sources. **(C)** Functional enrichment of DEGs. Glucose (MM), lignin (LM), cellulose (CM), xylan (XM), corncob (COM), cottonseed hull (CHM), wheat-straw (WSM), rice-straw (RSM), bran (BRM), pine wood sawdust (PWM), pine needle (PNM), spruce wood sawdust (SWM), spruce needle (SNM), pteridophyte (PTM), chestnut shell (CSM), chestnut leaf (CLM), bagasse (BM), oak wood sawdust (OWM), and potato dextrose agar (PDA).

The *G. lucidum* strain GL0102 was cultured on a total of 19 kinds of media with different carbon sources as solo carbon source (Table 1), respectively, and the growth rate was calculated accordingly. Mycelia cultured on media with glucose (MM), lignin (LM) or cellulose (CM) as carbon sources were sparse and had much lower growth rate (0.17 ± 0.004 to 0.37 ± 0.008 cm/d; Figure 1A; Supplementary Figure 1), while mycelia cultured on media with AFRs as carbon sources were lush and had higher growth rate, especially on wheat-straw (WSM), corncob (COM), pteridophyte (PTM), and chestnut leaf (CLM; 0.76 ± 0.015 to 0.80 ± 0.012 cm/d; Figure 1A; Supplementary Figure 1). This indicates that single-component carbon sources are possibly not comparable to complex carbon sources (AFRs) for mycelia production of *G. lucidum*.

To uncover the key genes involved in mycelia growth on different carbon sources, we compared the gene expression data obtained from the mycelia cultured on 18 carbon sources with that on glucose (MM), respectively. Among all the pairwise comparisons, 7 (XM vs. MM) to 1,865 (SNM vs. MM) genes were downregulated, while 16 (XM vs. MM) to 1,006 (RSM vs. MM) were upregulated (Figure 1B). According to their expression profile, all the DEGs could be divided into three main clusters (Supplementary Figure 2). Genes in cluster 1 showed relatively high expression on MM, XM, CM, and PDA; genes in cluster 2 showed relatively high expression on LM; and genes in cluster 3 showed relatively high expression on carbon sources derived from AFRs. Besides, the genes of these three clusters all showed enrichment in carbohydrate degradation-related functions, such as oxidoreductase activity, hydrolase activity, and carbohydrate metabolic process (Figure 1C).

Notably, the laccase gene family, which is closely related with lignin degradation, showed significant expression dynamics when the mycelia was cultured on different carbon sources. Thus, the laccase activity was tested. The mycelia cultured on single-component carbon source showed quite low laccase activity, such as 26 ± 7.3 U/mg on MM, 29 ± 13.1 U/mg on XM, and 32 ± 0.78 U/mg on CM (Figure 1A), while laccase activity of mycelia cultured on AFRs (except PTM) was significantly induced, showing values greater than 281 ± 35.4 U/mg (Figure 1A; Supplementary Figure 1). Specially, mycelia cultured on CSM showed laccase activity of 2,410 ± 122.4 U/mg which was much higher than those on other carbon sources (Figure 1A). Given that there are multiple laccase members in the *G. lucidum* genome, and the characteristics of each laccase member and their contribution to laccase activity may be different (Wang et al., 2015b; Ha et al., 2017; Kumar et al., 2017), comprehensive analysis regarding *G. lucidum* laccase was then performed.

### Genome-Wide Identification and Characterization of *Ganoderma lucidum* Laccase Genes

Totally, 15 and 16 laccase genes were identified in GL0102_53 and GL0102_8, respectively. According to the sequence similarity, two laccase genes in GL0102_8 were speculated to derived from duplication event. Hence, laccase genes in the two genomes were divided into 15 categories (*Gllac1-Gllac15*), and the two duplicated laccase genes in GL0102_8 were separately named as *H8lac7_1* and *H8lac7_2* (Supplementary Table 4). The laccase genes located on seven chromosomes of GL0102_8 and GL0102_53 and emerged the most frequently on chromosome 6 (Supplementary Table 4). Paired allelic laccase genes showed highly similar sequence features in protein length, pI, and MW. For example, *H8lac14* and *H53lac14* had minimal protein length of 460 aa, and *H8lac7_1* and *H53lac7* had maximal protein length of 667 aa (Supplementary Table 4). All the laccase genes were predicted to encode secreted proteins.

**Figure 2:**
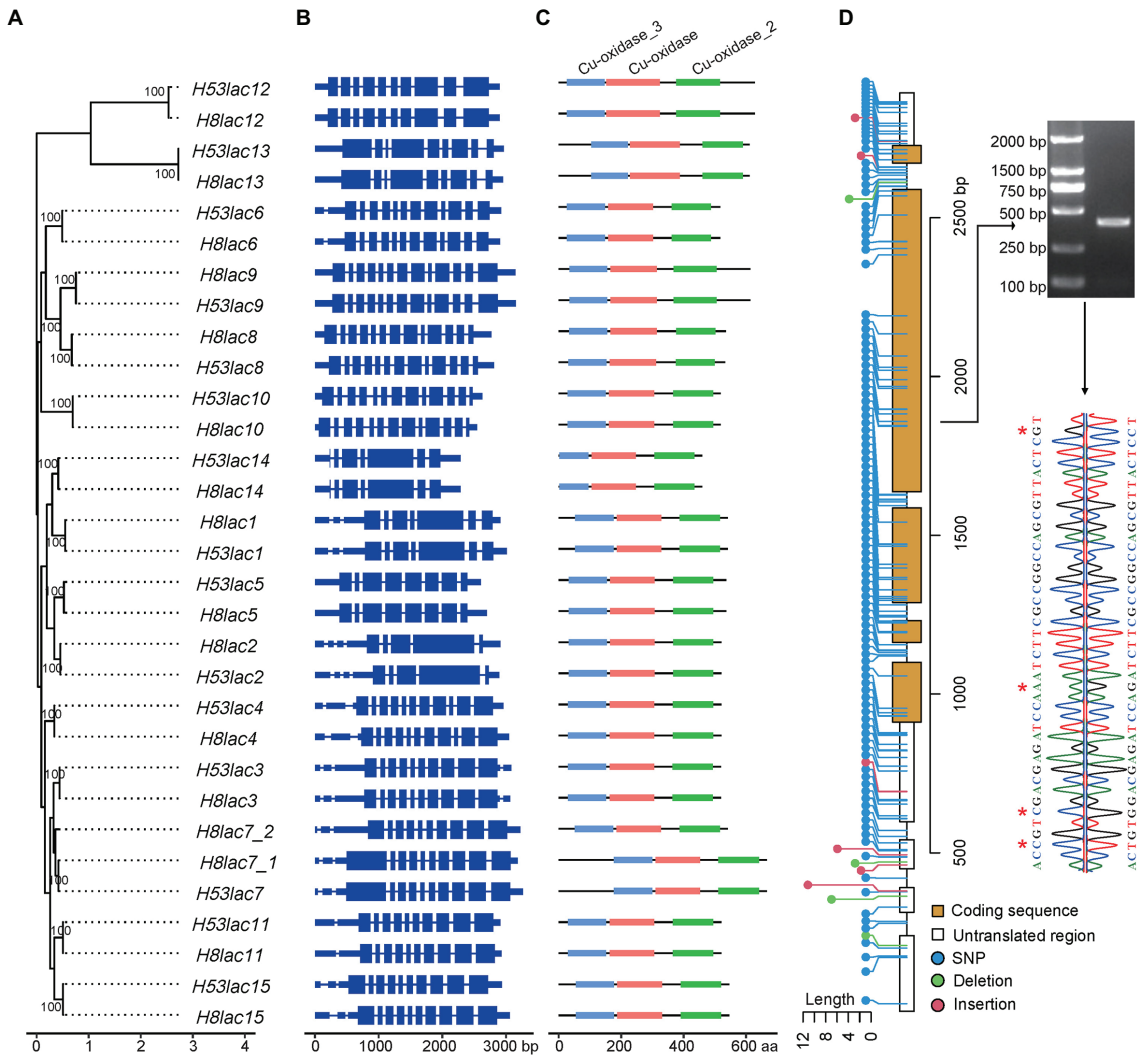
Characteristics of allelic laccase genes in *G. lucidum*. **(A)** Phylogenetic relationships (numbers on the nodes represent supporting values). **(B)** Gene structures. Blue rectangles represent the coding sequences, thin blue lines connecting two exons represent introns, and thick blue lines represent 5′-UTR or 3′-UTR. **(C)** Cu-domain. **(D)** Genetic variations, agarose gel electrophoresis, and Sanger sequencing of *Gllac2*. Red stars represent SNP loci.

A ML phylogenetic tree of laccase genes of GL0102 was constructed, and all paired allelic laccase genes clustered with each other (Figure 2A). It was reported that *G. lucidum* strain CGMCC5.0026 and P9 had 13 (Chen et al., 2012) and 16 (Liu et al., 2012) laccase genes, while 15 and 17 laccase genes were identified, respectively, after the manual correction in this study (*Gllac5* and *Gllac6* each had one more copy in P9; Supplementary Table 5). Besides, 15 laccase genes were identified in *G. lucidum* strain Lingjian-2 (Supplementary Table 5). In the phylogenetic tree constructed with all the above laccase genes, each of the 15 laccase categories from different strains/haploids were clustered together (Supplementary Figure 3), indicating that laccase genes were relatively conserved in *G. lucidum*.

As shown in Figure 2B, paired allelic laccases presented highly conserved gene structures and possessed relatively large number of introns, for example, each of *Gllac8*, *Gllac9*, and *Gllac10* had 12 introns. Each laccase contained three conserved domains: Cu-oxidase (PF00394.24), Cu-oxidase_2 (PF07731.16), and Cu-oxidase_3 (PF07732.17; Figure 2C).

Genetic variations were detected between paired allelic laccase genes, and SNPs were identified as the most abundant variations (Supplementary Table 6). Among all the allelic laccase pairs, 1 to 48 SNPs causing 0 to 16 amino acid changes were observed in coding regions, while none of the amino acid changes occurred in laccase signature sequence regions (Supplementary Table 6; Supplementary Figure 4). In general, the SNP rate was higher in untranslated regions (UTR) and introns. For example, a total of 113 SNPs and 10 insertions/deletions were identified in genic regions between *Gllac2* alleles, of which 42 SNPs existed in coding regions (Figure 2D). Randomly selected SNP loci between *H53lac2* and *H8lac2* were confirmed by Sanger sequence (Figure 2D).

### Expression Patterns of Laccase Genes on Different Carbon Sources

**Figure 3:**
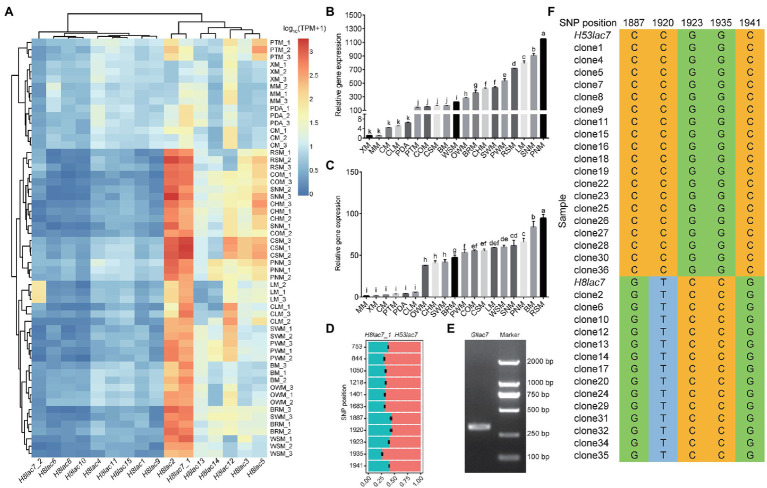
Expression patterns of laccase genes in *G. lucidum*. **(A)** Expression levels of 16 laccase genes on different carbon sources. **(B)** Relative expression of *Gllac2* detected by qPCR. **(C)** Relative expression of *Gllac7* detected by qPCR. **(D)** Read count of allelic *Gllac7* based on SNP loci in the mycelia cultured on RSM. **(E)** Agarose gel electrophoresis of PCR amplification products of partial *Gllac7* (311 bp). **(F)** SNP loci validation by clone sequencing. Glucose (MM), lignin (LM), cellulose (CM), xylan (XM), corncob (COM), cottonseed hull (CHM), wheat-straw (WSM), rice-straw (RSM), bran (BRM), pine wood sawdust (PWM), pine needle (PNM), spruce wood sawdust (SWM), spruce needle (SNM), pteridophyte (PTM), chestnut shell (CSM), chestnut leaf (CLM), bagasse (BM), oak wood sawdust (OWM), and potato dextrose agar (PDA). The error bars with different letters over the columns denote significant differences (*p* < 0.05).

The expression pattern of laccase genes on different carbon sources was further analyzed based on the RNA-Seq data using GL0102_8 genome as reference. Nine laccase genes (*Gllac1*, *Gllac4*, *Gllac6*, *Gllac8*, *Gllac9*, *Gllac10*, *Gllac11*, *Gllac15*, and *H8lac7_2*) showed quite low expression level (barely detectable, TPM value <10 under most conditions) under all the testing conditions (except that *H8lac7_2* showed relatively high expression level on LM), while the other seven laccase genes (*Gllac2*, *Gllac3*, *Gllac5*, *Gllac7*, *Gllac12*, *Gllac13* and *Gllac14*) showed high expression level especially on complex carbon sources (TPM value >100 under certain conditions; Figure 3A). Notably, *Gllac2* and *Gllac7* were the top two expressed laccase genes on complex carbon sources. Differential expression analysis was conducted on all carbon sources pairwisely. In all comparisons, 1 to 10 laccases were found differentially expressed. And the maximal number of differentially expressed laccase genes were found in the comparisons of XM vs. SNM and PDA vs. SNM (Supplementary Figure 5).

To test if the laccase expression was related with different growth stages, GL0102 mycelia cultured for 3, 5, and 7 days on MM, LM, CHM, RSM, BM, and PDA were collected, respectively. And the transcription levels of *Gllac2*, *Gllac4*, *Gllac7*, *Gllac9*, *Gllac10*, and *Gllac12* were analyzed by qPCR after we evaluated multiple parameters of the qPCR reaction and confirmed their acceptability (Supplementary Figure 6). Overall, the expression levels of *Gllac2* and *Gllac7* were highest in mycelia cultured for 3 days (on LM, RSM, BM and CHM) and decreased gradually with the increase of culture time (Supplementary Figure 7). Specifically, compared to 5d-mycelia cultured on MM, *Gllac2* showed 5-fold (on CLM) to 1,149-fold (on PNM) upregulation and *Gllac7* showed 4-fold (on PTM) to 94-fold (on RSM) upregulation on complex carbon sources (Figures 3B,C). *Gllac4*, *Gllac9* and *Gllac10* maintained low expression levels from 3 to 7 days (Supplementary Figure 7).

### Allelic Expression Bias Analysis of *Gllac7*

**Figure 4:**
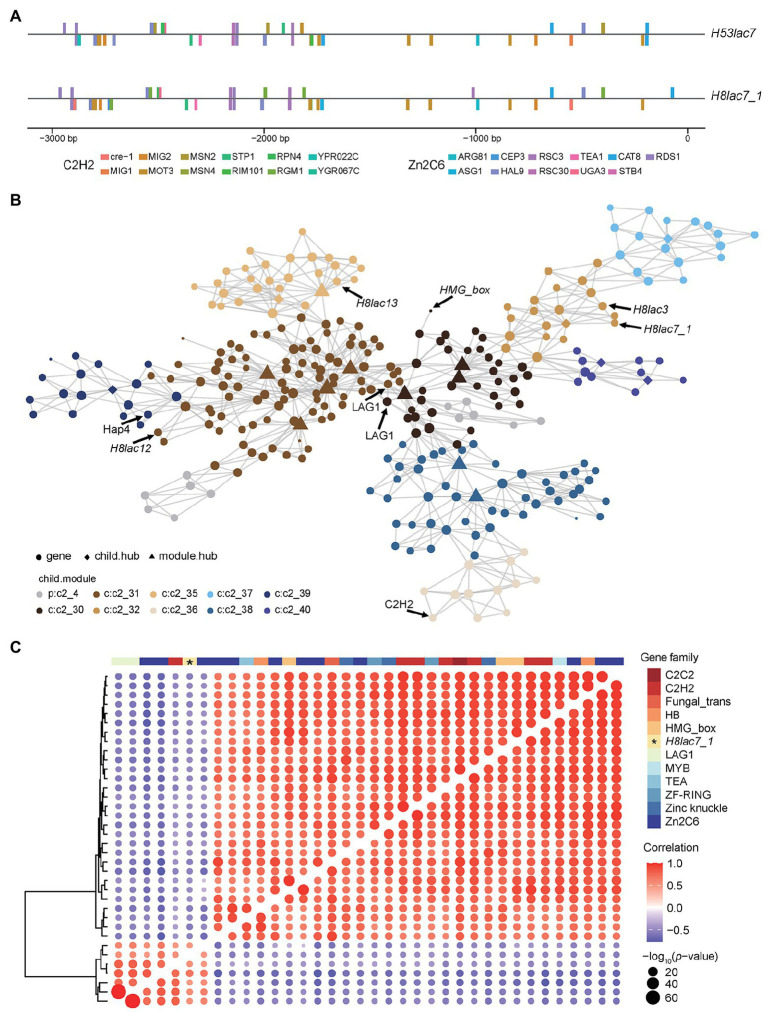
TF binding site analysis and co-expression network of *Gllac7*. **(A)** Predicted binding sites of zinc finger proteins in promoters of *H53lac7* and *H8lac7_1*. **(B)** The co-expression network of genes in the module containing *Gllac7*. **(C)** Correlation of *Gllac7* and TFs.

Further analysis showed that the two nuclei in *G. lucidum* contributed unevenly to the total expression of laccase, in other words, allelic expression bias was observed in laccase. On all the 19 carbon sources except XM, the expression of *H53lac7* was consistently higher than that of *H8lac7_1* (value of *p* < 0.05), and the read count ratio of *H53lac7* ranged from 51.71 to 76.54% with an average of 62.6% (Figure 3D; Supplementary Figure 8). To confirm this allelic bias case, partial sequences of *Gllac7* (311 bp), which contains SNP loci, were amplified using cDNA derived from RSM-cultured mycelia (Figure 3E). After cloning and sequencing, 19 out of 33 clones were consistent with *H53lac7,* while 14 clones were consistent with *H8lac7_1* (Figure 3F), confirming the existing of allelic expression bias. To analyze the possible causes of allelic expression bias, we analyzed the promoter regions of *H8lac7_1* and *H53lac7*. Totally, 47 SNPs and three insertions/deletions with length of 5, 8 and 9 bp were identified. These variants may cause differences in binding sites of transcription factors. For example, zinc fingers, a type of important regulators, have many binding sites in the promoter of *Gllac7*, while differences of binding sites were observed between promoters of *H53lac7* and *H8lac7_1* (Figure 4A).

A co-expression network was built based on expression levels of DEGs (in GL0102_8) using MEGENA pipeline (Song and Zhang, 2015). In the co-expression network, *Gllac7* (*H8lac7_1*), *H8lac3*, *H8lac12*, and *H8lac13* were clustered into a shared module (Figure 4B). This module contained 299 genes, among which five were TFs. Overall, there were 68 TFs in the DEGs and most TFs were downregulated on complex carbon sources. Moreover, Pearson correlation analysis between *Gllac7* and these TFs indicated that 35 TFs were significantly correlated with *Gllac7*, of which six TFs showed positive correlation, while the other 29 TFs showed negative correlation with *Gllac7* (Figure 4C). Most of these TFs belong to zinc finger family such as C2H2 (7/35) and Zn2C6 (12/35).

## Discussion

Carbon source affects the growth and metabolism of fungi (Wu et al., 2019; Lok et al., 2021; Zhou et al., 2021). *G. lucidum* has a relative wide carbon spectrum (Hapuarachchi et al., 2018; Kurd-Anjaraki et al., 2022), enabling it to be an ideal model fungus in research on carbon source utilization. AFRs, numerous in variety, are high quality biomass for *G. lucidum* cultivation. Thus, in this study, a total of 13 byproducts of agricultural (cottonseed hull, corncob, wheat-straw, rice-straw, bran, bagasse) and forestry (sawdust and leaf), and a widely distributed pteridophyte (*Dicranopteris pedata*) were used in mycelial cultivation of *G. lucidum*. Transcriptome analysis allows us to understand the overall impact of changes in carbon sources on gene expression network as a whole. In this study, up to 1,006 upregulation genes and 1,865 downregulation genes showed differential expression levels on divergent carbon sources (glucose as the control). Genes with function of oxidoreductase activity were significantly enriched, indicating their universal and indispensable roles in carbon utilization.

Due to its complex heterostructure, lignin becomes a natural antidegradation barrier of lignocellulose (Kumar and Chandra, 2020). Considering the vital function of laccase in lignin degradation, we conducted a comprehensive analysis toward laccase gene family of *G. lucidum*. Macro-fungi species usually contain more than 10 laccase genes, e.g., 14 in *Lentinula edodes* (Yan et al., 2019), 12 in *Pleurotus ostreatus* (Jiao et al., 2018), and 11 in *Flammulina velutipes* (Wang et al., 2015b). And it is assumed that the presence of large gene families of laccases in fungal genomes could be explained by broad spectrum of enzyme’s physiological functions required during the life cycle of a fungus (Kües and Rühl, 2011). In this study, 15 allelic laccase genes were identified in two haploid genomes (GL0102_8 and GL0102_53) of a *G. lucidum* strain GL0102, and *H8lac7_2* was possibly formed by gene duplication of *H8lac7_1* in GL0102_8. In addition, 15 laccase categories were confirmed in each of *G. lucidum* strain CGMCC5.0026, P9, and Lingjian-2 after a rigorously manual correction. The aforementioned results indicated that the 15 laccase categories in *G. lucidum* species are relatively conserved. Allelic laccase genes showed similar features (sequence characteristics/gene structures) and clustered together in the phylogenetic tree. Yet a certain number of genetic variations, especially SNPs, were identified in paired allelic laccase genes, indicating genetic diversity of allelic laccase genes in *G. lucidum*. Although SNPs caused several amino acid changes between allelic laccases of *G. lucidum*, none of the changes were located in conserved domain. More SNPs are located in non-coding regions which may have substantial impact on gene regulation, thereby contributing to phenotypic diversity (Albert and Kruglyak, 2015; van Arensbergen et al., 2019).

Although a relatively large number of laccase genes were existed in macro-fungi, only one or a few laccase isozymes showed high expression abundance (Ha et al., 2017), and a few laccases showed activity in the Native-PAGE under one or some given conditions (Wang et al., 2015a; Kumar et al., 2017). In this study, laccase activity of mycelia cultured on LM was medium, less than that on AFRs. Compared to AFRs, the ingredients of commercial lignin (LM) are more homogeneous. The lignin with complex structures and categories in AFRs was considered to have stronger induction for laccase activity. Laccases may have functional differentiation and different members under fine regulation. Thus, the relatively large laccase gene members may be a result of function redundancy, or the appropriate expression conditions for some laccase genes have not been revealed. Enhanced understanding of genetic mechanisms of laccase expression and regulation would benefit for optimal laccase production in *G. lucidum* using biotechnologies. In this study, more than half (9) of the *G. lucidum* laccase genes showed quite low expression level under all the testing conditions, while the other seven laccase genes showed obviously higher expression level in complex carbon sources than in single-component carbon sources. The induced gene expression may contribute to the fast growth and high laccase activity of *G. lucidum* mycelia cultured on complex carbon sources. Among all the laccase genes, *Gllac2* and *Gllac7* showed maximal expression abundance on complex carbon sources especially in the early stage of mycelial culture, indicating their inducible transcription by exogenous polysaccharides.

In diploid basidiomycetes, two nuclei co-exist in the common cytoplasm but divide independently (Clark and Anderson, 2004). Thus, whether one of the nuclei is dominant in allelic gene expression is worthy exploring. To date, studies on allele-specific gene expression have mainly focused on humans or animals (Tian et al., 2018; Tangwancharoen et al., 2020), with only a few cases having been reported in fungi. According to Ha *et al*, expression bias in the allelic laccase genes of two nuclei of *L. edodes* was detected (Ha et al., 2017). In this study, allelic expression bias was found in *Gllac7*, and the expression of *H53lac7* was consistently higher than that of *H8lac7_1* under the testing conditions (except on XM). The coordination of allelic genes in the expression may be a result of environmental adaptation, and allelic expression bias may contribute to formation of physiological characteristics of a nucleus, which will be useful in elite strain selection.

In carbon metabolism, zinc finger is a vital regulator, and the C2H2 zinc finger CreA has been widely identified as a universal carbon catabolism repressor (Kunitake et al., 2019; Hu et al., 2020). And in *G. lucidum*, *PacC*, a C2H2 zinc finger was proved to negatively regulate laccase activity (Zhu et al., 2022). In this study, zinc finger TFs showed significant correlation with expression of *Gllac7*, and many binding sites of zinc finger proteins were detected in the *Gllac7* promoter, indicating their potential regulation roles of *Gllac7*.

## Data Availability Statement

The datasets presented in this study can be found in online repositories. The names of the repository/repositories and accession number(s) can be found at: http://www.gpgenome.com/species/408.

## Author Contributions

LW, LL, and QW conceived the project. QH, BH, XD, and QW performed the experiments. LW and QW analyzed the data. LW drafted the manuscript. LW and XD revised the manuscript. All authors contributed to the article and approved the submitted version.

## Funding

This research was funded by GDAS’ Project of Science and Technology Development (2020GDASYL-20200103071 and 2022GDASZH-2022010110), Guangzhou Science and Technology Program Project (202103000080), Special Support Program of Guangdong Province (2019TQ05N232), and Heyuan Science and Technology Program Project (HYZP201909007).

## Conflict of Interest

The authors declare that the research was conducted in the absence of any commercial or financial relationships that could be construed as a potential conflict of interest.

## Publisher’s Note

All claims expressed in this article are solely those of the authors and do not necessarily represent those of their affiliated organizations, or those of the publisher, the editors and the reviewers. Any product that may be evaluated in this article, or claim that may be made by its manufacturer, is not guaranteed or endorsed by the publisher.

## Footnotes

^1^ http://www.bioinformatics.babraham.ac.uk/projects/fastqc/

^2^ http://www.gpgenome.com/species/408

^3^ https://cran.rproject.org/web/packages/pheatmap/

^4^ http://www.ebi.ac.uk/Tools/pfa/pfamscan

^5^ https://web.expasy.org/protparam/

^6^ http://www.cbs.dtu.dk/services/SignalP/

^7^ http://pfam.xfam.org/

^8^ http://jaspar.genereg.net/

^9^ http://www.ciiider.org/
