# Supplementary material for: Gllac7 Is Induced by Agricultural and Forestry Residues and Exhibits Allelic Expression Bias in Ganoderma lucidum
Source: Front Microbiol. 2022 Jun 30;13:890686. doi: 10.3389/fmicb.2022.890686 (PMC9279560; doi:10.3389/fmicb.2022.890686)
Supplement: Supplementary file 1 [file Data_Sheet_1.DOCX]

**Supplementary Table 1** Statistics of RNA quality

| **Sample** | **Conc**  **(ng/μL)** | **OD260/**  **280** | **OD260/**  **230** | **25S/**  **18S** | **RIN** | **Sample** | **Conc**  **(ng/μL)** | **OD260/**  **280** | **OD260/**  **230** | **25S/**  **18S** | **RIN** |
| --- | --- | --- | --- | --- | --- | --- | --- | --- | --- | --- | --- |
| MM_1 | 3620 | 2.2 | 2.1 | 1.4 | 8.8 | PNM_1 | 1515 | 2.2 | 2.2 | 1.6 | 9.3 |
| MM_2 | 2065 | 2.2 | 2.1 | 1.5 | 8.9 | PNM_2 | 759 | 2.2 | 2.3 | 1.6 | 8.9 |
| MM_3 | 1620 | 2.2 | 2.0 | 1.5 | 9.9 | PNM_3 | 1900 | 2.2 | 2.2 | 1.5 | 9.0 |
| LM_1 | 2330 | 2.2 | 2.2 | 2.3 | 8.3 | SWM_1 | 1720 | 2.2 | 2.2 | 1.8 | 9.5 |
| LM_2 | 5380 | 2.0 | 2.0 | 2.4 | 7.8 | SWM_2 | 1875 | 2.2 | 2.3 | 1.6 | 9.5 |
| LM_3 | 2890 | 2.2 | 2.0 | 1.7 | 9.3 | SWM_3 | 1330 | 2.2 | 2.3 | 1.7 | 9.7 |
| CM_1 | 1770 | 2.2 | 2.3 | 1.4 | 8.5 | SNM_1 | 2400 | 2.2 | 2.1 | 1.5 | 8.9 |
| CM_2 | 1450 | 2.2 | 2.3 | 1.4 | 8.1 | SNM_2 | 2340 | 2.2 | 2.2 | 1.3 | 8.2 |
| CM_3 | 1995 | 2.2 | 2.3 | 1.5 | 9.1 | SNM_3 | 2485 | 2.2 | 2.2 | 1.6 | 10.0 |
| XM_1 | 3560 | 2.0 | 2.0 | 1.5 | 10.0 | PTM_1 | 1846 | 2.2 | 2.3 | 1.7 | 8.8 |
| XM_2 | 4670 | 1.9 | 1.8 | 1.5 | 9.7 | PTM_2 | 2155 | 2.2 | 2.3 | 1.6 | 9.3 |
| XM_3 | 1340 | 2.2 | 2.3 | 1.5 | 8.2 | PTM_3 | 3890 | 2.2 | 2.2 | 1.4 | 8.3 |
| COM_1 | 4940 | 2.2 | 2.2 | 1.4 | 8.5 | CSM_1 | 305 | 1.8 | 1.6 | 1.7 | 9.4 |
| COM_2 | 2550 | 2.2 | 2.2 | 1.4 | 8.2 | CSM_2 | 461 | 1.7 | 1.4 | 1.7 | 8.9 |
| COM_3 | 2950 | 2.2 | 2.2 | 1.4 | 8.2 | CSM_3 | 477 | 1.8 | 1.4 | 1.7 | 9.8 |
| CHM_1 | 2870 | 2.2 | 2.3 | 1.7 | 9.3 | CLM_1 | 987 | 2.0 | 1.7 | 1.4 | 9.2 |
| CHM_2 | 1035 | 2.2 | 2.2 | 1.7 | 9.6 | CLM_2 | 509 | 1.9 | 1.7 | 1.4 | 9.4 |
| CHM_3 | 2195 | 2.2 | 2.3 | 1.7 | 9.7 | CLM_3 | 1310 | 2.0 | 1.8 | 1.5 | 9.9 |
| WSM_1 | 1083 | 2.2 | 2.3 | 1.6 | 9.3 | BM_1 | 2650 | 2.2 | 2.3 | 1.5 | 8.1 |
| WSM_2 | 2680 | 2.2 | 2.3 | 1.6 | 9.0 | BM_2 | 3710 | 2.2 | 2.2 | 1.8 | 7.8 |
| WSM_3 | 3540 | 2.2 | 2.3 | 1.6 | 9.5 | BM_3 | 2220 | 2.2 | 2.3 | 2.2 | 8.2 |
| RSM_1 | 4350 | 2.2 | 2.1 | 1.6 | 9.3 | OWM_1 | 3190 | 2.2 | 2.2 | 1.5 | 7.8 |
| RSM_2 | 1790 | 2.2 | 2.0 | 1.8 | 9.6 | OWM_2 | 1630 | 2.2 | 2.1 | 1.5 | 7.9 |
| RSM_3 | 2485 | 2.2 | 2.0 | 1.8 | 9.6 | OWM_3 | 2450 | 2.2 | 2.2 | 1.4 | 8.1 |
| BRM_1 | 4130 | 2.2 | 2.3 | 1.5 | 9.0 | PDA_1 | 4660 | 2.1 | 1.9 | 1.6 | 10.0 |
| BRM_2 | 3580 | 2.2 | 2.3 | 1.5 | 9.0 | PDA_2 | 3960 | 2.1 | 2.1 | 1.7 | 10.0 |
| BRM_3 | 2680 | 2.2 | 2.3 | 1.7 | 9.1 | PDA_3 | 1524 | 2.1 | 2.0 | 1.5 | 9.7 |
| PWM_1 | 3550 | 2.2 | 2.3 | 1.6 | 9.5 |  |  |  |  |  |  |
| PWM_2 | 2275 | 2.2 | 2.3 | 1.6 | 9.3 |  |  |  |  |  |  |
| PWM_3 | 3300 | 2.2 | 2.3 | 1.6 | 8.8 |  |  |  |  |  |  |

Conc represents concentration of RNA, RIN represents RNA Intergrity Number. Glucose (MM), lignin (LM), cellulose (CM), xylan (XM), corncob (COM), cottonseed hull (CHM), wheat-straw (WSM), rice-straw (RSM), bran (BRM), pine wood sawdust (PWM), pine needle (PNM), spruce wood sawdust (SWM), spruce needle (SNM), pteridophyte (PTM), chestnut shell (CSM), chestnut leaf (CLM), bagasse (BM), oak wood sawdust (OWM), and potato dextrose agar (PDA).

**Supplementary Table 2** Statistics of transcriptome sequencing reads after quality control

| **Sample** | **readNumber** | **baseNumber** | **GCcontent** | **Q20** | **Q30** | **Sample** | **readNumber** | **baseNumber** | **GCcontent** | **Q20** | **Q30** |
| --- | --- | --- | --- | --- | --- | --- | --- | --- | --- | --- | --- |
| CSM-1 | 44147312 | 6622096800 | 59.9684 | 98.0323 | 94.5211 | CHM-1 | 44924312 | 6738646800 | 59.9041 | 98.3349 | 94.5942 |
| CSM-2 | 45119854 | 6767978100 | 59.8836 | 98.3706 | 95.491 | CHM-2 | 45601698 | 6840254700 | 58.8071 | 98.7437 | 95.8716 |
| CSM-3 | 46473810 | 6971071500 | 60.0091 | 97.8488 | 94.0111 | CHM-3 | 45427214 | 6814082100 | 59.8519 | 98.7653 | 96.0243 |
| CLM-1 | 39468856 | 5920328400 | 60.3245 | 98.3927 | 94.7749 | PDA_1 | 44873064 | 6730959600 | 59.6648 | 98.9224 | 97.0007 |
| CLM-2 | 44020878 | 6603131700 | 60.0444 | 98.7194 | 95.9207 | PDA_2 | 40470614 | 6070592100 | 59.5293 | 98.8887 | 96.9823 |
| CLM-3 | 45926776 | 6889016400 | 59.9232 | 98.4746 | 95.071 | PDA_3 | 47992420 | 7198863000 | 59.6119 | 98.8065 | 96.7515 |
| BM_1 | 40649334 | 6097400100 | 58.8806 | 98.8291 | 96.8596 | SNM-1 | 45422658 | 6813398700 | 59.66 | 98.4583 | 94.8875 |
| BM_2 | 46172720 | 6925908000 | 59.603 | 98.8639 | 97.0046 | SNM-2 | 46654484 | 6998172600 | 60.0285 | 98.2004 | 94.1646 |
| BM_3 | 45470050 | 6820507500 | 59.9824 | 98.7566 | 96.6835 | SNM-3 | 46330492 | 6949573800 | 60.113 | 98.7367 | 95.8777 |
| CM_1 | 44907970 | 6736195500 | 58.4044 | 98.9151 | 97.0763 | PWM-1 | 54432918 | 8164937700 | 59.8094 | 98.3629 | 95.0433 |
| CM_2 | 41809248 | 6271387200 | 59.1311 | 98.7519 | 96.7392 | PWM-2 | 44385012 | 6657751800 | 60.1041 | 98.4657 | 95.0574 |
| CM_3 | 45436930 | 6815539500 | 59.3146 | 98.7596 | 96.8016 | PWM-3 | 64329526 | 9649428900 | 60.0832 | 97.9896 | 93.9509 |
| RSM-1 | 46264294 | 6939644100 | 56.7336 | 98.7678 | 95.978 | SWM-1 | 47081912 | 7062286800 | 59.9562 | 98.4507 | 94.9902 |
| RSM-2 | 44136562 | 6620484300 | 60.2226 | 98.5752 | 95.4382 | SWM-2 | 46063102 | 6909465300 | 59.3751 | 98.4063 | 94.8426 |
| RSM-3 | 45468922 | 6820338300 | 57.659 | 98.1915 | 94.0794 | SWM-3 | 47974964 | 7196244600 | 59.8603 | 98.4437 | 94.9589 |
| BRM-1 | 43155610 | 6473341500 | 53.7347 | 97.679 | 92.6473 | PNM-1 | 45384136 | 6807620400 | 59.7425 | 98.5439 | 95.2609 |
| BRM-2 | 39891326 | 5983698900 | 59.3817 | 98.4021 | 94.8005 | PNM-2 | 44625762 | 6693864300 | 59.8578 | 97.9462 | 93.388 |
| BRM-3 | 42771550 | 6415732500 | 59.9088 | 98.2118 | 94.1822 | PNM-3 | 47025568 | 7053835200 | 59.772 | 97.882 | 93.1697 |
| LM_1 | 42721692 | 6408253800 | 60.3141 | 98.8824 | 96.9764 | OWM_1 | 41082402 | 6162360300 | 59.9315 | 98.8219 | 96.8925 |
| LM_2 | 44900112 | 6735016800 | 60.2263 | 98.7972 | 96.8583 | OWM_2 | 43337070 | 6500560500 | 59.9228 | 98.7091 | 96.612 |
| LM_3 | 46718200 | 7007730000 | 60.3407 | 98.8047 | 96.7803 | OWM_3 | 46156590 | 6923488500 | 59.985 | 98.6679 | 96.479 |
| PTM-1 | 44565446 | 6684816900 | 59.5903 | 98.3772 | 94.7813 | XM_1 | 40054532 | 6008179800 | 59.6869 | 98.7701 | 96.6988 |
| PTM-2 | 46223348 | 6933502200 | 59.812 | 98.6237 | 95.5251 | XM_2 | 45411398 | 6811709700 | 59.7727 | 98.6923 | 96.4973 |
| PTM-3 | 44208906 | 6631335900 | 59.6711 | 98.4687 | 95.0638 | XM_3 | 44802256 | 6720338400 | 59.7198 | 98.8511 | 96.9082 |
| WSM-1 | 45460902 | 6819135300 | 59.8997 | 98.6506 | 95.6116 | COM-1 | 44819582 | 6722937300 | 59.6932 | 98.2134 | 94.2014 |
| WSM-2 | 60501722 | 9075258300 | 58.023 | 98.3426 | 94.8964 | COM-2 | 45837428 | 6875614200 | 59.4576 | 98.5194 | 95.229 |
| WSM-3 | 45822092 | 6873313800 | 59.8273 | 98.6532 | 95.6403 | COM-3 | 52481208 | 7872181200 | 58.0685 | 98.7533 | 96.109 |
| MM_1 | 46595310 | 6989296500 | 59.8047 | 98.5901 | 96.3057 |  |  |  |  |  |  |
| MM_2 | 44744750 | 6711712500 | 59.6165 | 98.7288 | 96.5881 |  |  |  |  |  |  |
| MM_3 | 44020308 | 6603046200 | 59.7436 | 98.4466 | 96.02 |  |  |  |  |  |  |

**Supplementary Table 3** Primer sequences used in this study

| **Primer name** | **Sequence**  **(5’→3’)** | **Length**  **(bp)** | **Application** |
| --- | --- | --- | --- |
| Gllac2_F | GAATGAGCAAGTTCTCGTCCTT | 250 | qPCR of *Gllac2* |
| Gllac2_R | GCGGTCAACATGGTAGAGTTAG |  |  |
| Gllac7_F | GTCGTGGTCAATGGTGTCTTC | 121 | qPCR of *Gllac7* |
| Gllac7_R | AGTGGATGCTGGTGGTCTTC |  |  |
| gapdh_F | GGTGCCAAGAAGGTGGTCAT | 139 | qPCR of reference gene |
| gapdh_R | CGAGAGGAGCCAGACAGTTG |  |  |
| Gllac4_F | TTGACGGCACATACTTCTACAC | 228 | qPCR of *Gllac4* |
| Gllac4_R | GGACCACAGCGAACGAATG |  |  |
| Gllac9_F | CTCTGATCCTGTACGACAAGAAC | 113 | qPCR of *Gllac9* |
| Gllac9_R | TGGCAACAACGGAGCGATA |  |  |
| Gllac10_F | GACGAGAGCACGATCATCAC | 148 | qPCR of *Gllac10* |
| Gllac10_R | CGACCGTAATCACCGACAAG |  |  |
| Gllac12_F | CGATTGGTATCACGACGAACA | 146 | qPCR of *Gllac12* |
| Gllac12_R | GGTTGGTGCCGCTAATTGG |  |  |
| Gllac2_407_F | CTGGTCTCCATGTCGTGTGA | 407 | Validation of SNP loci in *Gllac2* |
| Gllac2_407_R | GTGCCGTTGAAGGTGAAGG |  |  |
| Gllac7_311_F | GTTCCATCTTCACGGTCAC | 311 | Validation of *Gllac7* allelic expression bias |
| Gllac7_311_R | ACTGGTCATCGGGAGAGA |  |  |

**Supplementary Table 4** Characteristics of laccase genes of *G. lucidum*

| **Name** | **Protein length**  **(amino acid)** | **Isoelectric**  **point** | **Molecular weight**  **(kDa)** | **Signal peptide** | **Distribution** |
| --- | --- | --- | --- | --- | --- |
| *Gllac1* | 541 / 541 | 4.94 / 4.89 | 57.68 / 57.54 | yes/yes | chr1/chr1 |
| *Gllac2* | 521 / 521 | 4.5 / 4.47 | 55.27 / 55.28 | yes/yes | chr1/chr1 |
| *Gllac3* | 520 / 520 | 5.58 / 5.66 | 56.17 / 56.17 | yes/yes | chr3/chr3 |
| *Gllac4* | 521 / 521 | 4.82 / 4.78 | 55.76 / 55.72 | yes/yes | chr3/chr3 |
| *Gllac5* | 537 / 537 | 4.5 / 4.5 | 57.88 / 57.88 | yes/yes | chr3/chr3 |
| *Gllac6* | 517 / 517 | 5.45 / 5.72 | 56.45 / 56.42 | yes/yes | chr6/chr6 |
| *Gllac7* | 667 /667, 542 | 5.67 / 5.67, 4.89 | 72.76 / 72.73, 58.64 | yes/yes, yes | chr6/chr6, chr6 |
| *Gllac8* | 533 / 535 | 5.17 / 5.12 | 58.00 / 58.21 | yes/yes | chr6/chr6 |
| *Gllac9* | 614 / 614 | 6.38 / 6.38 | 67.30 / 67.30 | yes/yes | chr6/chr6 |
| *Gllac10* | 519 / 519 | 5.15 / 5.03 | 56.92 / 56.91 | yes/yes | chr6/chr6 |
| *Gllac11* | 521 / 521 | 5.54 / 5.54 | 57.15 / 57.17 | yes/yes | chr8/chr11 |
| *Gllac12* | 628 / 628 | 5.01 / 4.97 | 68.31 / 68.39 | yes/yes | chr9/chr9 |
| *Gllac13* | 611 / 611 | 5.12 / 5.16 | 65.82 / 65.76 | yes/yes | chr11/chr11 |
| *Gllac14* | 460 / 460 | 4.8 / 4.85 | 49.86 / 49.95 | yes/yes | chr11/chr11 |
| *Gllac15* | 546 / 546 | 5.22 / 5.22 | 59.07 / 59.09 | yes/yes | chr13/chr13 |

The data to the left and right of the ‘/’ represents values in GL0102_53 and GL0102_8, respectively. In the line starting with *Gllac7*, the data to the right of the ‘/’ represents values in *H8lac7_1* and *H8lac7_2*, respectively.

**Supplementary Table 5** Laccase genes in different strains or haploids of *G. lucidum*

| **Laccase** | **CGMCC**  **5.0026** | **P9** | **Lingjian-2** | **GL0102_8** | **GL0102_53** |
| --- | --- | --- | --- | --- | --- |
| *Gllac1* | *5.0026lac1* | *P9lac1* | *Lingjian2lac1* | *H8lac1* | *H53lac1* |
| *Gllac2* | *5.0026lac2* | *P9lac2* | *Lingjian2lac2* | *H8lac1* | *H53lac2* |
| *Gllac3* | *5.0026lac3* | *P9lac3* | *Lingjian2lac3* | *H8lac1* | *H53lac3* |
| *Gllac4* | *5.0026lac4* | *P9lac4* | *Lingjian2lac4* | *H8lac1* | *H53lac4* |
| *Gllac5* | *5.0026lac5* | *P9lac5_1 / P9lac5_2* | *Lingjian2lac5* | *H8lac1* | *H53lac5* |
| *Gllac6* | *5.0026lac6* | *P9lac6_1 / P9lac6_2* | *Lingjian2lac6* | *H8lac1* | *H53lac6* |
| *Gllac7* | *5.0026lac7* | *P9lac7* | *Lingjian2lac7* | *H8lac7_1 / H8lac7_2* | *H53lac7* |
| *Gllac8* | *5.0026lac8* | *P9lac8* | *Lingjian2lac8* | *H8lac1* | *H53lac8* |
| *Gllac9* | *5.0026lac9* | *P9lac9* | *Lingjian2lac9* | *H8lac1* | *H53lac9* |
| *Gllac10* | *5.0026lac10* | *P9lac10* | *Lingjian2lac10* | *H8lac1* | *H53lac10* |
| *Gllac11* | *5.0026lac11* | *P9lac11* | *Lingjian2lac11* | *H8lac1* | *H53lac11* |
| *Gllac12* | *5.0026lac12* | *P9lac12* | *Lingjian2lac12* | *H8lac1* | *H53lac12* |
| *Gllac13* | *5.0026lac13* | *P9lac13* | *Lingjian2lac13* | *H8lac1* | *H53lac13* |
| *Gllac14* | *5.0026lac14* | *P9lac14* | *Lingjian2lac14* | *H8lac1* | *H53lac14* |
| *Gllac15* | *5.0026lac15* | *P9lac15* | *Lingjian2lac15* | *H8lac1* | *H53lac15* |

**Supplementary Table 6** Genetic variations between paired allelic laccase genes

| **Laccase** | **GL0102_8** | **GL0102_53** | **Genetic variations in genic region** | | | **SNP in coding region** | **Number of changed amino acids** |
| --- | --- | --- | --- | --- | --- | --- | --- |
|  |  |  | **Insertion** | **Deletion** | **SNP** |  |  |
| *Gllac1* | *H8lac1* | *H53lac1* | 12 | 11 | 123 | 34 | 16 |
| *Gllac2* | *H8lac1* | *H53lac2* | 6 | 4 | 113 | 42 | 4 |
| *Gllac3* | *H8lac1* | *H53lac3* | 0 | 1 | 15 | 1 | 1 |
| *Gllac4* | *H8lac1* | *H53lac4* | 1 | 0 | 59 | 14 | 1 |
| *Gllac5* | *H8lac1* | *H53lac5* | 0 | 1 | 33 | 19 | 1 |
| *Gllac6* | *H8lac1* | *H53lac6* | 3 | 6 | 92 | 31 | 6 |
| *Gllac7* | *H8lac7_1* | *H53lac7* | 0 | 3 | 48 | 14 | 3 |
| *Gllac8* | *H8lac1* | *H53lac8* | 0 | 8 | 37 | 11 | 2 |
| *Gllac9* | *H8lac1* | *H53lac9* | 4 | 4 | 26 | 7 | 0 |
| *Gllac10* | *H8lac1* | *H53lac10* | 0 | 1 | 42 | 25 | 3 |
| *Gllac11* | *H8lac1* | *H53lac11* | 0 | 0 | 2 | 2 | 1 |
| *Gllac12* | *H8lac1* | *H53lac12* | 0 | 2 | 15 | 6 | 2 |
| *Gllac13* | *H8lac1* | *H53lac13* | 0 | 2 | 7 | 1 | 1 |
| *Gllac14* | *H8lac1* | *H53lac14* | 1 | 0 | 81 | 48 | 12 |
| *Gllac15* | *H8lac1* | *H53lac15* | 0 | 0 | 46 | 10 | 4 |


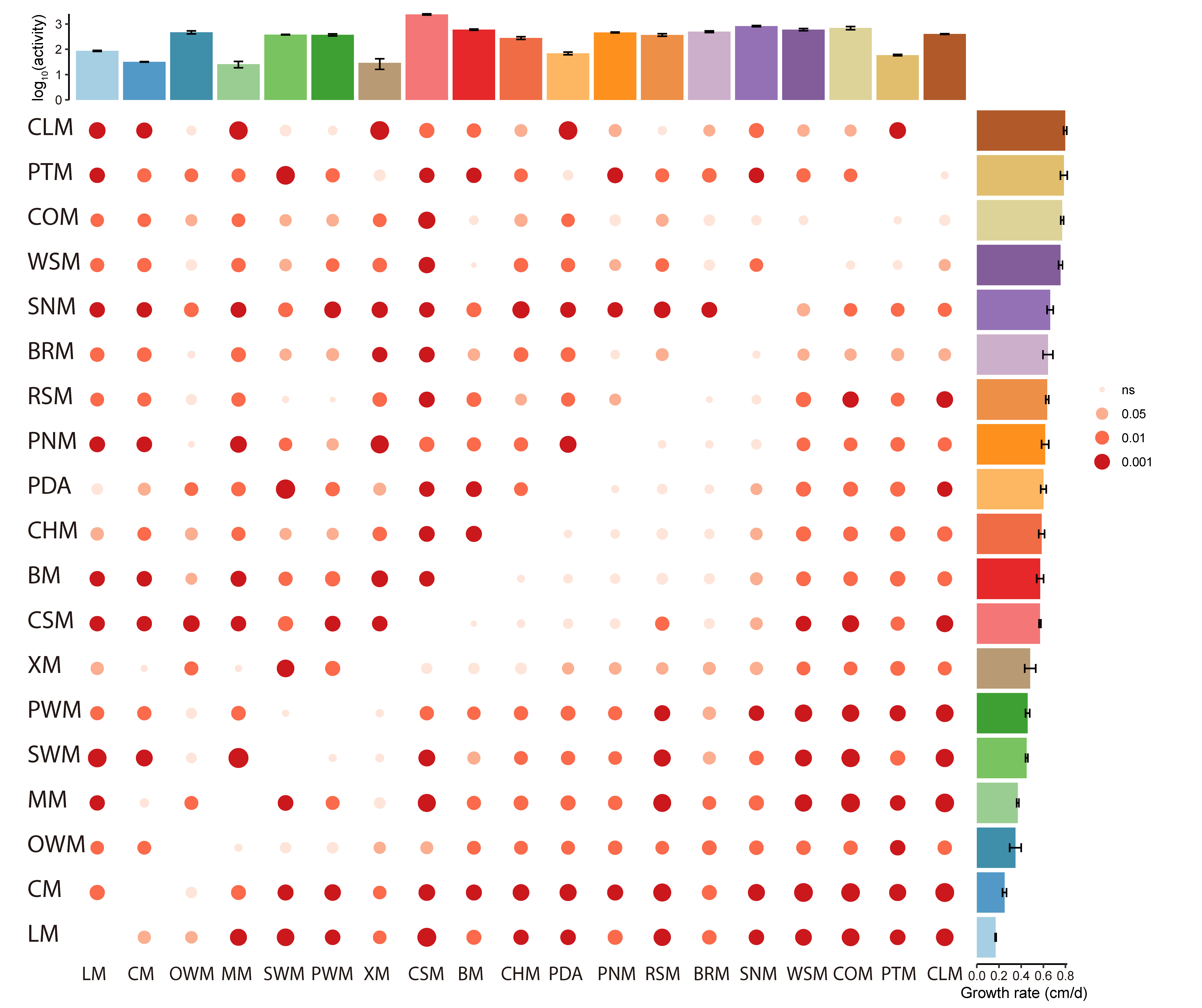


**Supplementary Figure 1** Significant difference analysis of laccase activity and growth rate of mycelia cultured on different carbon sources. Glucose (MM), lignin (LM), cellulose (CM), xylan (XM), corncob (COM), cottonseed hull (CHM), wheat-straw (WSM), rice-straw (RSM), bran (BRM), pine wood sawdust (PWM), pine needle (PNM), spruce wood sawdust (SWM), spruce needle (SNM), pteridophyte (PTM), chestnut shell (CSM), chestnut leaf (CLM), bagasse (BM), oak wood sawdust (OWM), and potato dextrose agar (PDA).


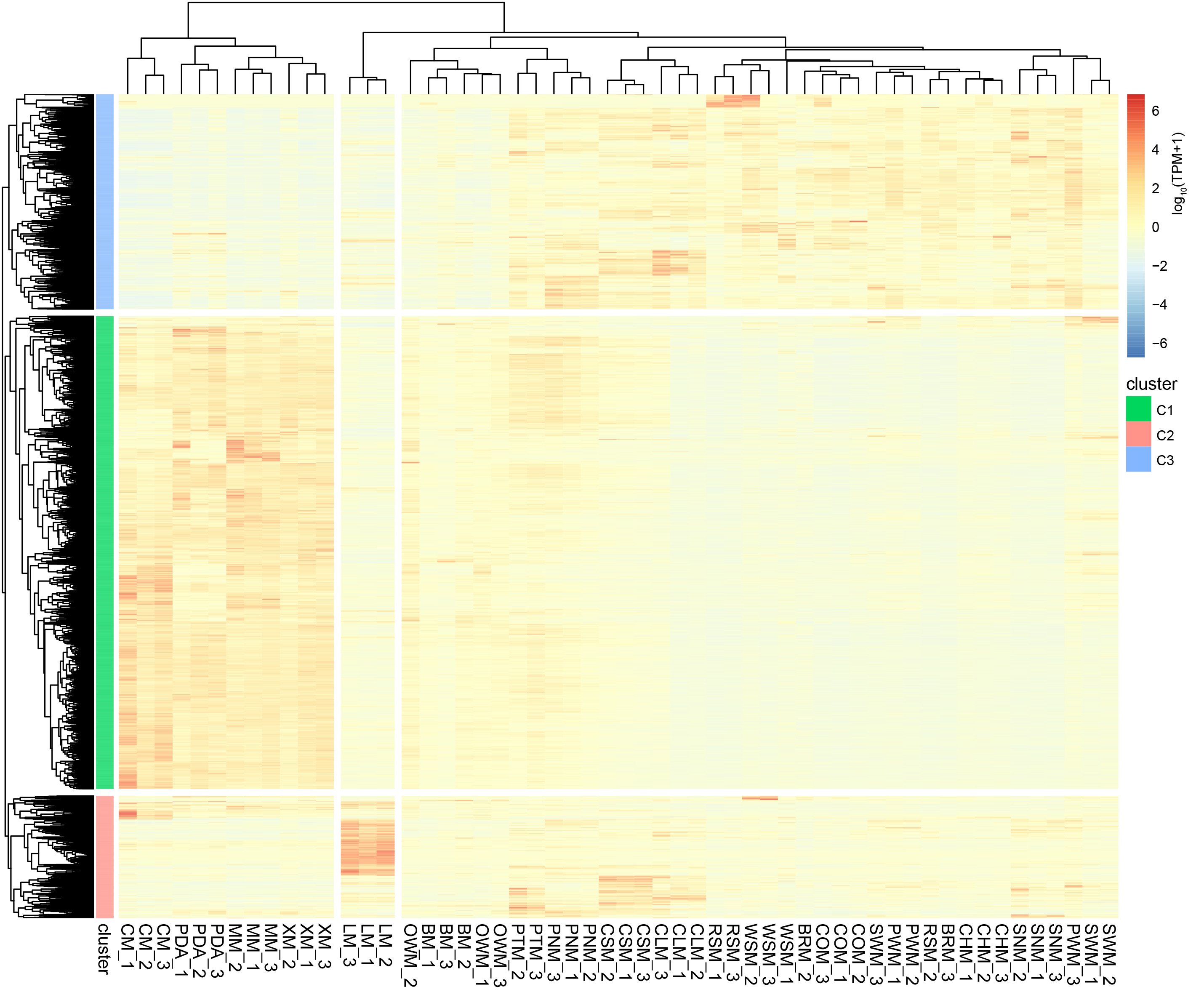


**Supplementary Figure 2** Expression heatmap of differentially expressed genes under different conditions. Glucose (MM), lignin (LM), cellulose (CM), xylan (XM), corncob (COM), cottonseed hull (CHM), wheat-straw (WSM), rice-straw (RSM), bran (BRM), pine wood sawdust (PWM), pine needle (PNM), spruce wood sawdust (SWM), spruce needle (SNM), pteridophyte (PTM), chestnut shell (CSM), chestnut leaf (CLM), bagasse (BM), oak wood sawdust (OWM), and potato dextrose agar (PDA).


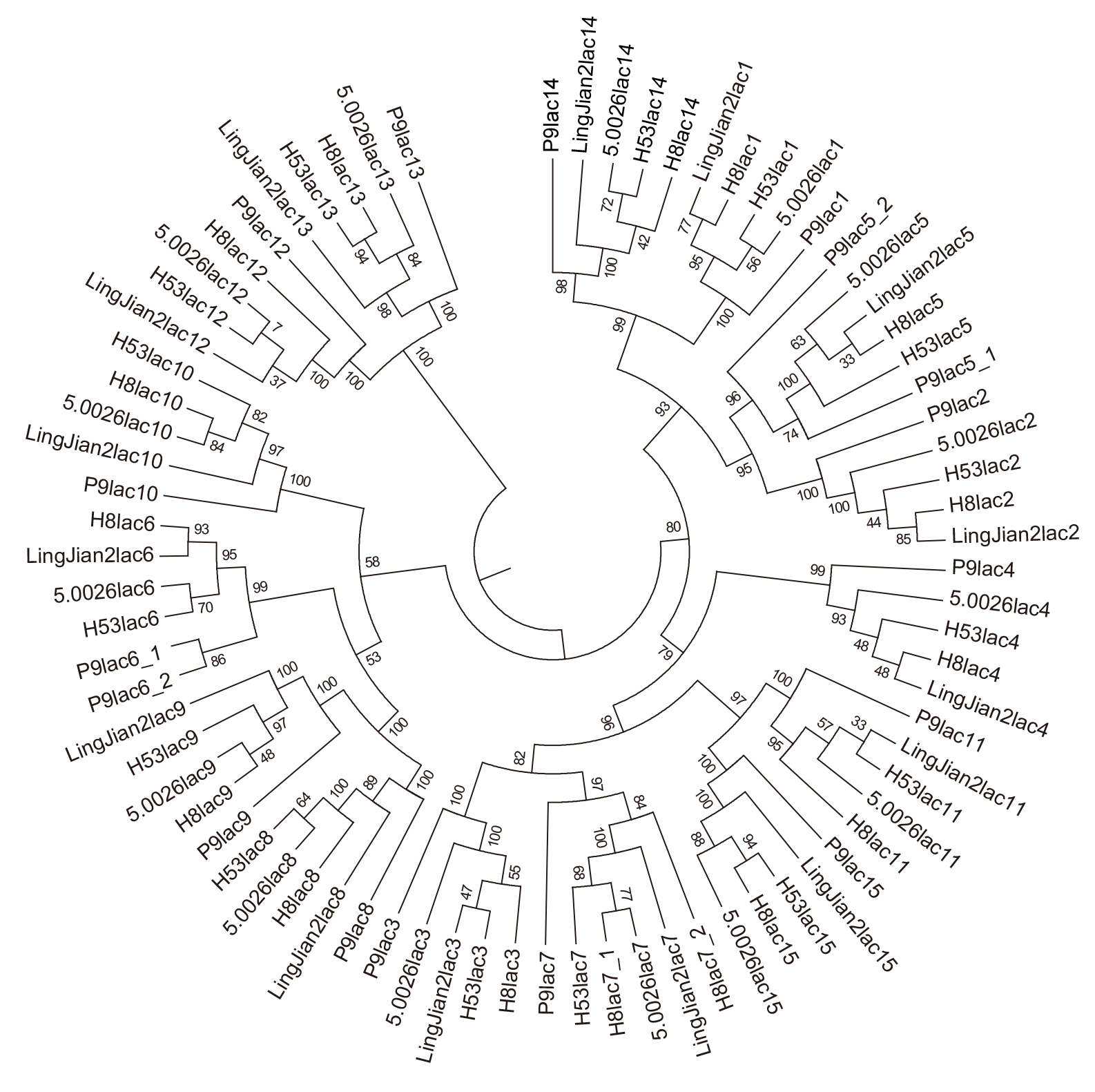


**Supplementary Figure 3** Phylogenetic tree of 78 *G. lucidum* laccase genes. Numbers on the branch represent bootstrap supporting values.


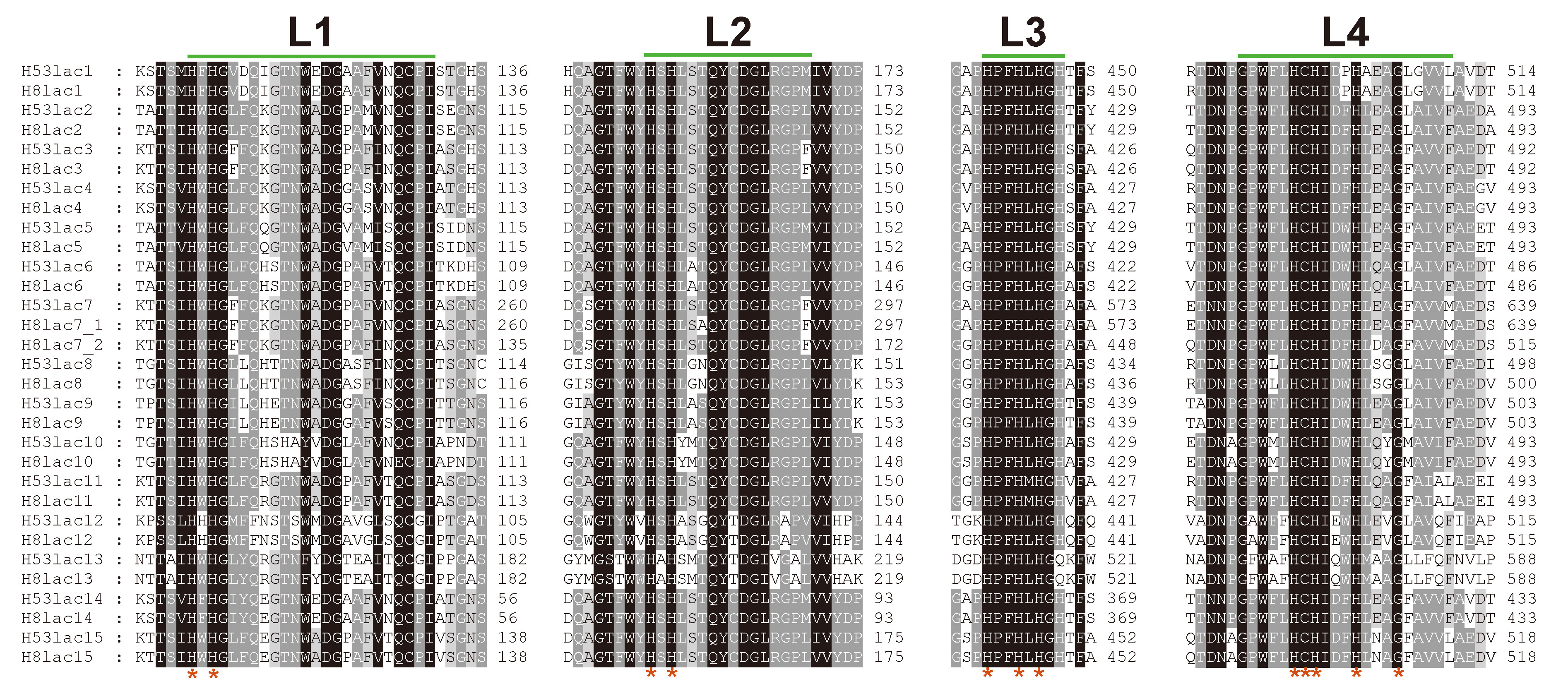


**Supplementary Figure 4** Multiple sequence alignment of laccases of *G. lucidum*. L1–L4 represent typical laccase signature sequences, red asterisks indicate copper ligands.


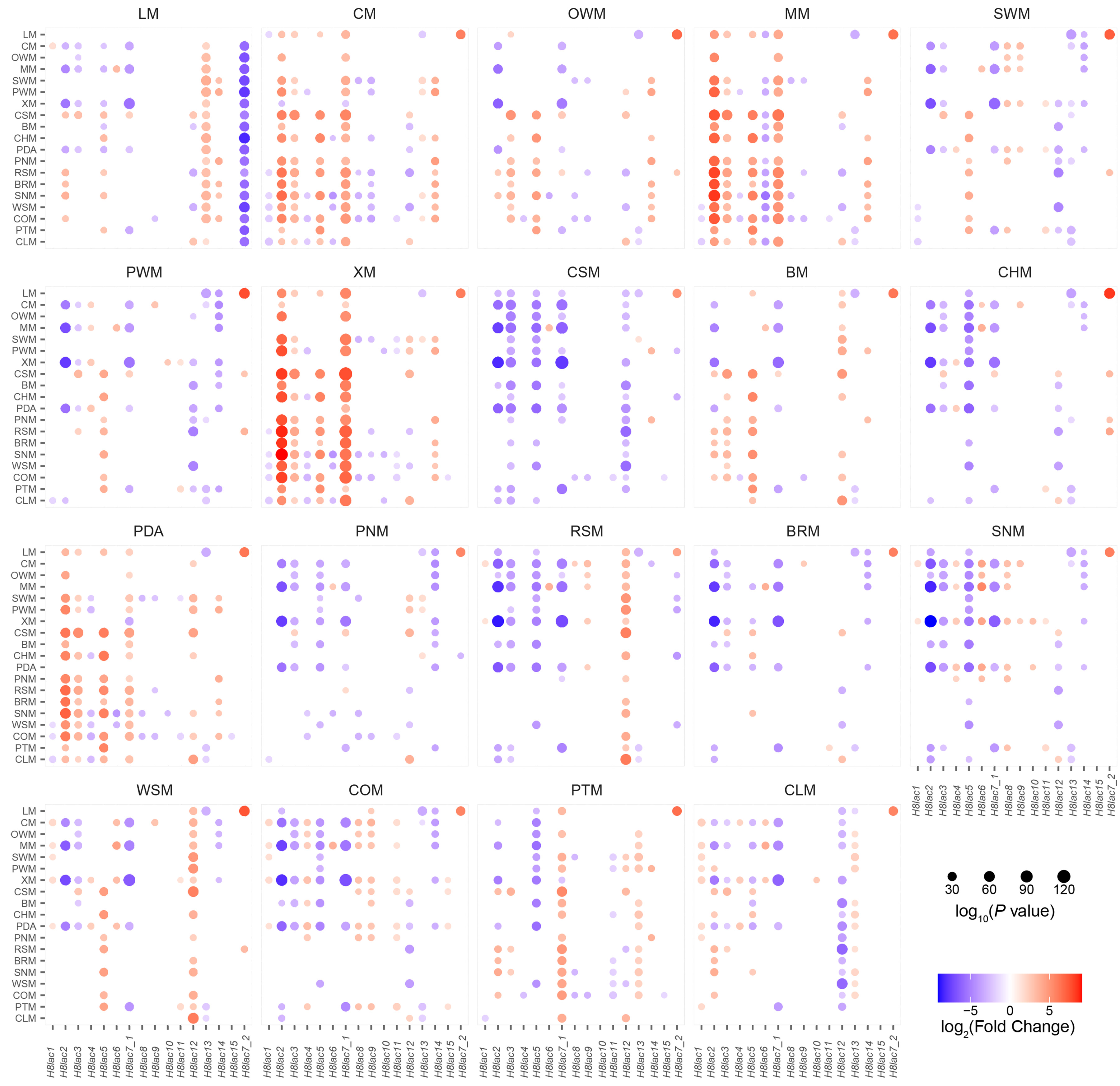


**Supplementary Figure 5** Differential expression of *G. lucidum* laccase genes among different carbon sources.


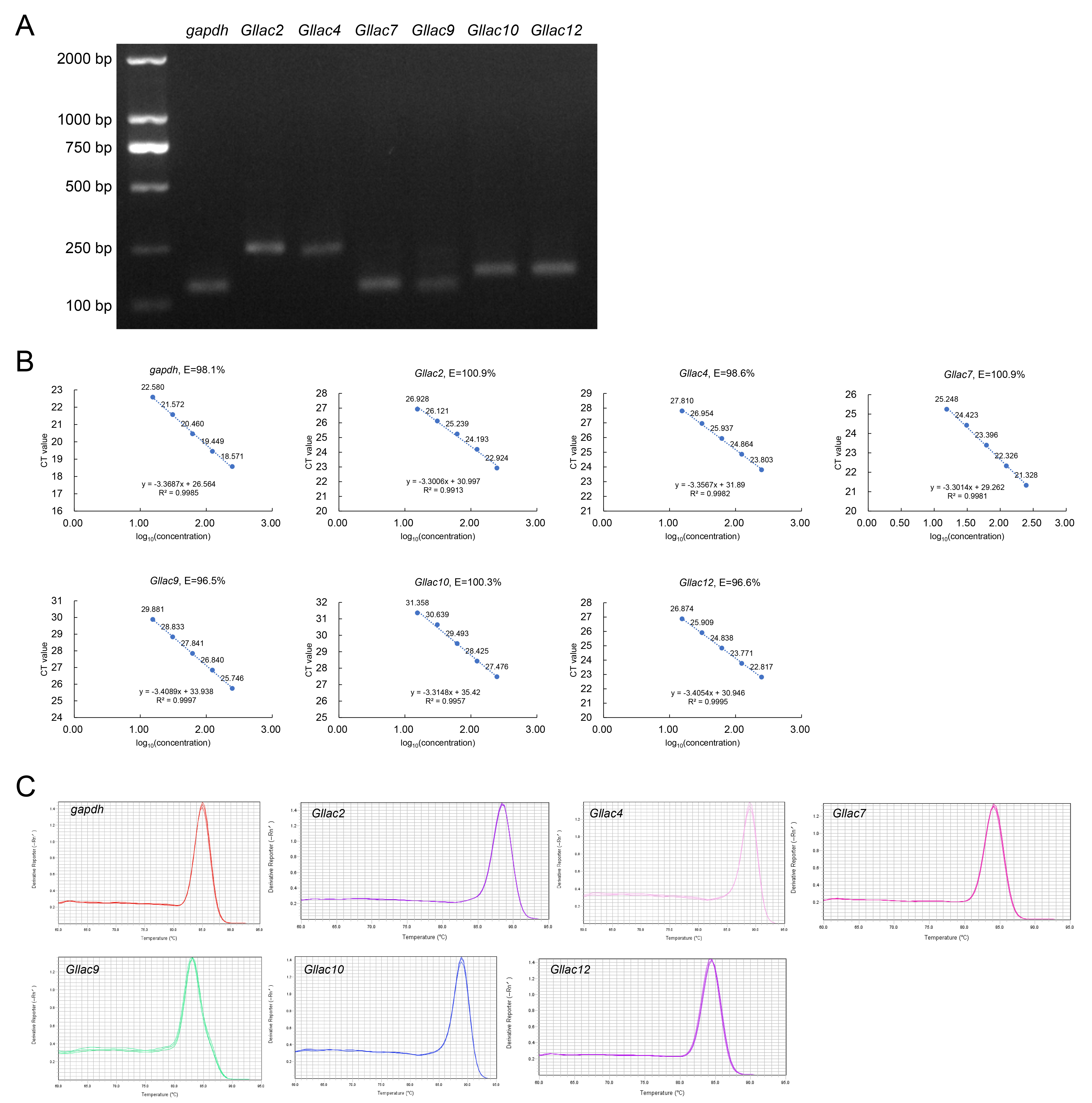


**Supplementary Figure 6** Parameters of qPCR reaction. (A) Agarose gel electrophoresis of qPCR products; (B) Standard curve; (C) melt curve.


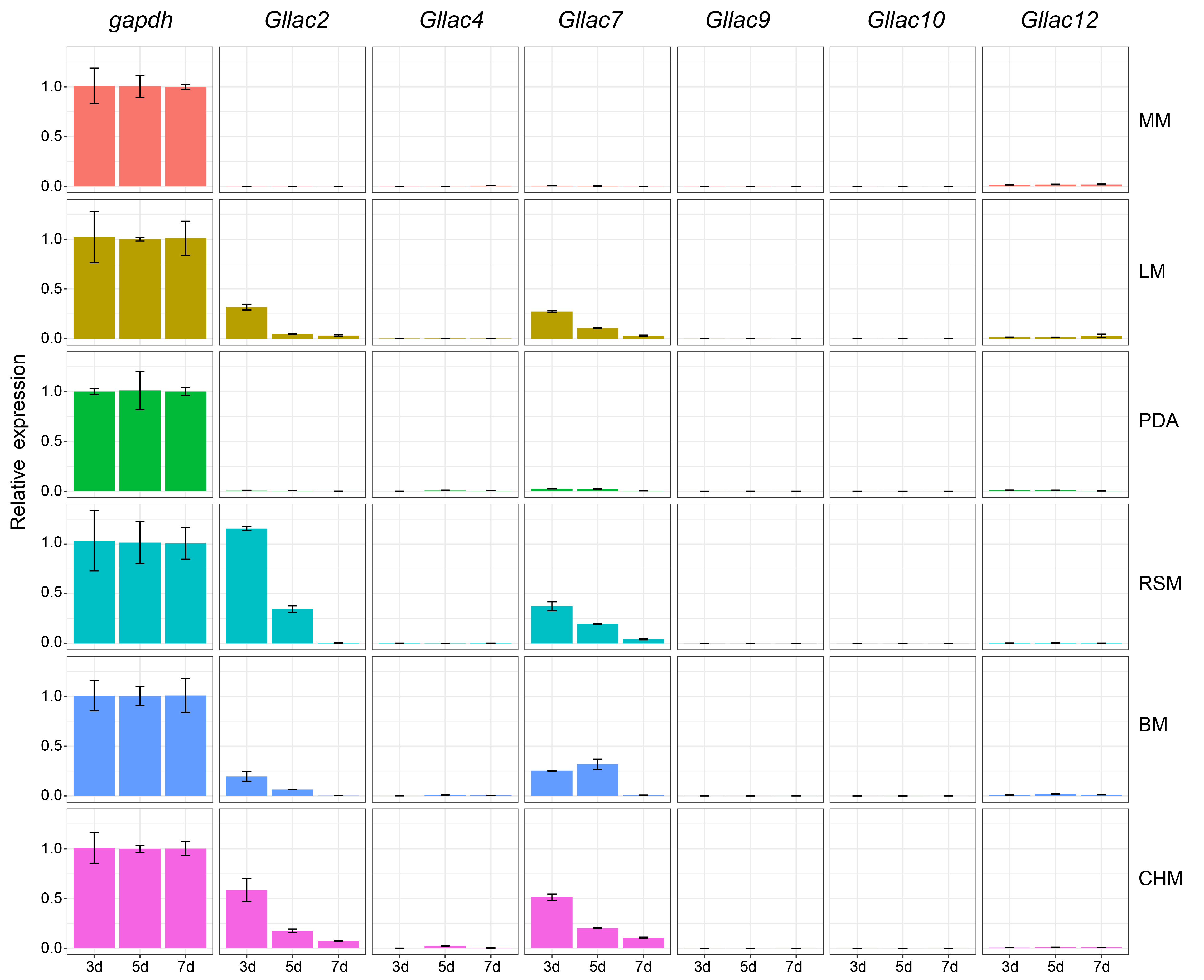


**Supplementary Figure 7** Expression of laccase genes at different growth stages. Glucose (MM), lignin (LM), cottonseed hull (CHM), rice-straw (RSM), bagasse (BM), and potato dextrose agar (PDA).


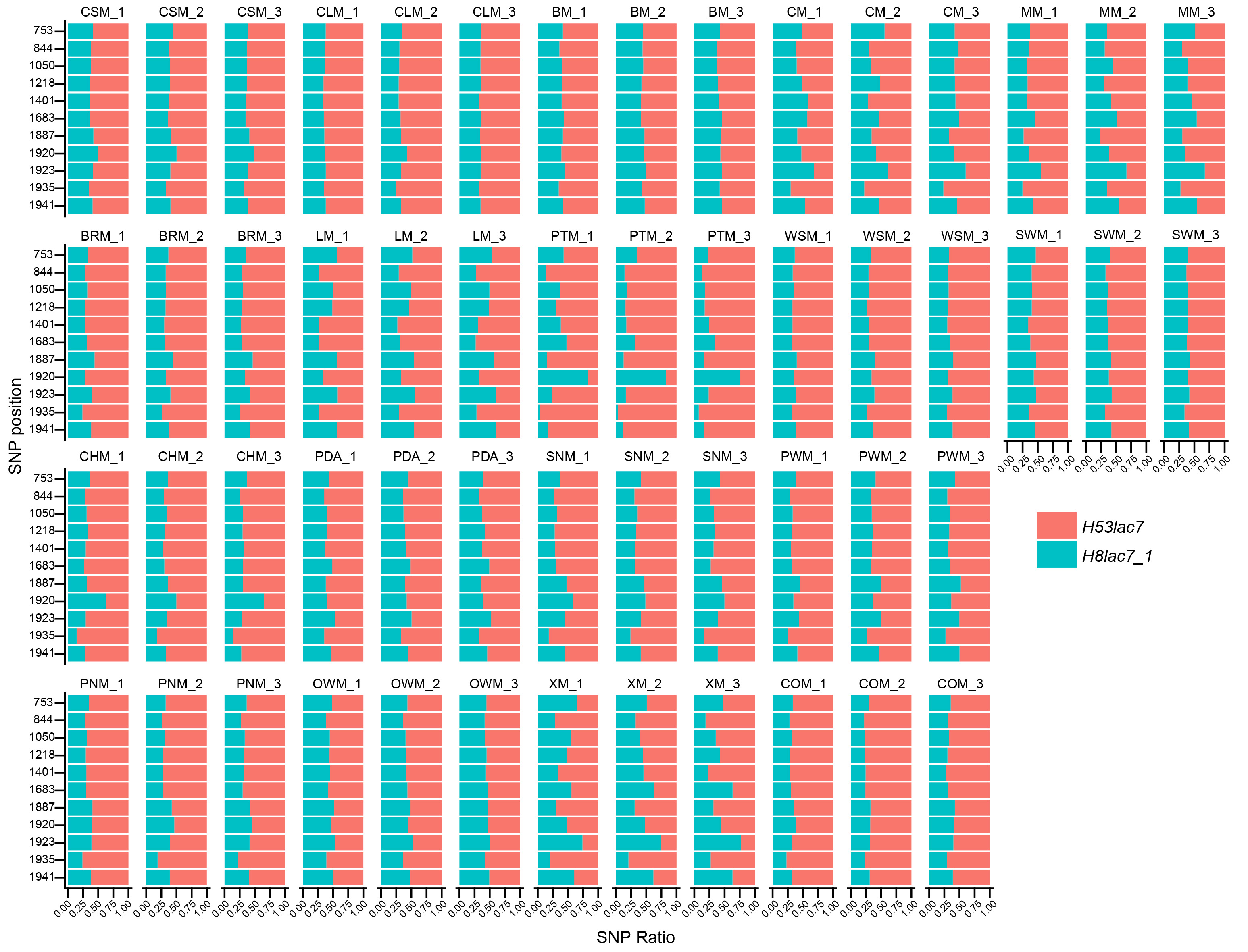


**Supplementary Figure 8** Allelic expression bias of *Gllac7* under different conditions. Glucose (MM), lignin (LM), cellulose (CM), xylan (XM), corncob (COM), cottonseed hull (CHM), wheat-straw (WSM), bran (BRM), pine wood sawdust (PWM), pine needle (PNM), spruce wood sawdust (SWM), spruce needle (SNM), pteridophyte (PTM), chestnut shell (CSM), chestnut leaf (CLM), bagasse (BM), oak wood sawdust (OWM), and potato dextrose agar (PDA).
